# Healthcare adjustments and concerns: a qualitative study exploring the perspectives of healthcare providers and administrative staff during the COVID-19 pandemic in Saudi Arabia

**DOI:** 10.3389/fpubh.2023.961060

**Published:** 2023-05-11

**Authors:** Mohammed S. Alkathlan, Yasir A. Alsuyufi, Abdulhamid F. Alresheedi, Rehana Khalil, Parveen Anjum Sheiq, Suliman S. Alotaieq, Abdullah A. Almithn, Ibrahim I. Alissa, Hamad F. Alayyaf, Raed M. Alharbi, Ibrahim A. Alkhamis, Osama Al-Wutayd

**Affiliations:** ^1^MD Consultant Infectious Diseases, King Fahad Specialist Hospital, Buraydah, Saudi Arabia; ^2^MD Consultant Pediatric Gastroenterologist, King Saud Hospital, Unaizah, Saudi Arabia; ^3^MD Critical Care Consultant, Hayat National Hospital, Unaizah, Saudi Arabia; ^4^Department of Family and Community Medicine, Unaizah College of Medicine and Medical Sciences, Qassim University, Unaizah, Saudi Arabia; ^5^Department of Basic Medical Sciences, Unaizah College of Medicine and Medical Sciences, Qassim University, Unaizah, Saudi Arabia; ^6^Medical Students, Unaizah College of Medicine and Medical Sciences, Qassim University, Unaizah, Saudi Arabia

**Keywords:** healthcare, adjustments, concerns, qualitative study, Saudi Arabia, COVID-19, pandemic

## Abstract

**Background:**

Healthcare systems have modified their strategies to manage their staff, supplies, and space to deal systematically with the COVID-19 pandemic. This research aimed to explore the nature of hospital adjustments and the concerns of healthcare providers and administrative staff working in Governmental and private hospitals throughout the Qassim Region of the Kingdom of Saudi Arabia (KSA) during the pandemic.

**Methods:**

A qualitative phenomenological study using semi-structured in-depth interviews were conducted with 75 purposively selected healthcare providers and administrative staff working at three main hospitals in the Qassim Region, KSA. The maximum variation sampling technique was utilized. Recruitment of participants was continued until data saturation was reached. All interviews were audiotaped, transcribed verbatim, and analyzed thematically.

**Results:**

Four core themes were identified in this paper: (1) changes in hospital policy and procedures, (2) workforce management, (3) the well-being of the workforce, and (4) apprehensions and expectations of the workforce. The participants showed satisfaction with timely administrative decisions and new policies during the COVID-19 pandemic. Furthermore, the psychological health of healthcare professionals was affected more than their physical state. Finally, the providers perceived the emergence of multiple concerns in the coming months.

**Conclusion:**

Although healthcare providers were initially overwhelmed, they gradually accepted new administrative policies. Numerous innovative interventions effectively reduced their physical workload and increased their productivity, but they remained significantly affected by a wide range of psychological disorders, with a high prevalence of obsessive-compulsive disorder. There were some concerns about the new SARS-CoV-2 variant, but the majority were optimistic.

## Introduction

As the COVID-19 pandemic enters its second year, it has considerably shaped our lives with immense lockdowns and requirements for social distancing and wearing facemasks (1). Although most countries worldwide have already vaccinated their adult populations, they continue to face challenges brought about by new strains of the virus, competition over limited vaccine supplies, and vaccine hesitation (2). The pandemic has also stretched national healthcare systems to their limits (3). To deal systematically with this unprecedented challenge, hospitals worldwide have modified their strategies to manage their staff, supplies, and space for the provision of optimum care to their patients (4). At the same time, almost every country in the world has experienced an enormous exhaustion of hospital resources and infection of healthcare workers (5). A study conducted in China concluded that in the initial phase of the COVID-19 pandemic in the country, the lack of efficient protective measures led to the emotional stress and psychological disturbance of the majority of the hospital staff; fortunately, the lessons they learned from such experiences helped them respond to the outbreak in later phases (6). Another study identified a need to adapt new modes of operation and to implement suitable modifications to patient care policies to deal with the challenges of this pandemic (7). Similar findings have been reported in a contemporary study, which concluded that (1) a multi-level and multidisciplinary approach by healthcare professionals and hospital administrators is needed toward the common goal of patient care, and (2) the protocols need revision on a regular basis to assure the best possible patient care (8). A study conducted in 10 countries, including two low-income countries e.g., Ethiopia and Haiti, six middle-income e.g., Ghana, Lao People's Democratic Republic, Mexico, Nepal, South Africa and Thailand, and high-income e.g., Chile and South Korea, to evaluate the effect of the COVID-19 pandemic on 31 healthcare services (9). The authors found interruptions of different magnitude and duration in different services in all studied countries, and they didn't found any patterns by country's income or intensity of pandemic. Various reasons have been identified for decreased healthcare use during COVID-19 pandemic, which include the people's fear of getting infected while visiting the health facilities, the cancellation or suspension of non-COVID-19 related care and the barriers due to lockdown policies (10, 11). The healthcare system of the Kingdom of Saudi Arabia (KSA) is considered one of the most organized systems in the world, with a World Health Organization (WHO) ranking of 26th out of 191 countries (12). The KSA lies between the Red Sea and the Arabian Gulf and is the largest country in the Arabian Peninsula (13). It has a total population of 34,813,871, and consists of 13 provinces (14). The KSA was among the first countries to immediately respond to and implement disease containment measures proactively when the WHO instructed countries to control the spread of the COVID-19 virus through the adoption of an organized, methodical approach (15, 16). The country currently has 494 hospitals, 113,000 physicians, and a ratio of 22.5 beds/10,000 people (14, 17). The Ministry of Health (MoH) is responsible for the provision of healthcare services to all residents (17). Studies have highlighted the fact that the policies that were put in place against the Middle East Respiratory Syndrome (MERS) outbreaks were extremely helpful in the country's understanding of public health priorities during large-scale outbreaks of communicable diseases, such as COVID-19 (18, 19). To deal with the COVID-19 pandemic, the MoH established a national emergency response committee and activated its command-and-control center for the continuous monitoring of national and international updates, health surveillance, population screening, contact tracing, and raising public awareness of COVID-19 (20). The National Health Command Center (NHCC) dashboard, which was initially set up to monitor the day-to-day performance of primary healthcare and hospital facilities through a series of apps and other digital systems, was eventually adapted as an early-warning system for the MoH staff to keep tabs on every progress against COVID-19 throughout the KSA (15). These digital tools enable data sharing, equitable access to health services, and improved coordination (21, 22). The MoH also established laboratory testing facilities to provide advanced diagnostic services, and biosafety in diagnostic laboratories and infection control systems was improved substantially in all hospitals across the kingdom (15, 20, 23). Recognizing the importance of timely health interventions for policy actions, our study aimed to explore the newly implemented regulations at Governmental and private hospitals located in the Qassim Region, KSA, during the current pandemic, as well as the experiences and concerns of healthcare providers and administrative staff serving at those hospitals.

## Methods

### Study design

A qualitative study with phenomenological approach of in-depth interviews was conducted, including both healthcare providers and administrative staff to get a comprehensive view at different levels on the role they played as policy maker or implementer of new hospital protocols, and to recount their observations and practices as they tried to comply with the new hospital policies and regulations during the global pandemic crisis (24, 25). This approach allowed the researchers to understand the healthcare providers and administrative staff's perspectives (26). The in-depth interviews were conducted using a semi-structured guide, in which the participants were encouraged to shape the discussion in accordance with their experiences, views, and concerns (27).

### Study setting

Three (two governmental and one private) hospitals of two big cities of Al Qassim region were selected to have all-inclusive approach toward changes and challenges encountered during pandemic. Two (one governmental and one private hospital) out of three were located in the city of Unaizah, the second largest city, with a population of approximately 165,000. The city lies in the central region of the KSA, called the Qassim region. King Saud Hospital (KSH) is a governmental hospital with a capacity of around 294 beds (28, 29). KSH is one of 25 regional hospitals designated by the MoH for the isolation and treatment of COVID-19 patients (15). The second hospital was Hayat National Hospital (HNH), a private hospital with 130 beds in 16 specialties (30). The third hospital was King Fahad Specialist Hospital (KFSH), a 430-bed tertiary care hospital located in Buraidah City, the largest city and the capital of the Qassim Region, with a population of approximately 619,739 (28, 29).

### Procedure

#### Identification and recruitment of participants

To gain conversance of a range of experiences, we recruited a sample of administrative staff and healthcare providers working in all the representative departments/units of three main (governmental and private) hospitals of the Qassim region using a maximum variation sampling technique (31). The recruitment phase started with an organized and systematized identification of potential participants, followed by the preparation of the lists of identified participants and their basic information. Healthcare providers and administrators were considered eligible if they met the following inclusion criteria for surgeons, physicians, lab assistants, nurses, and hospital administrators: (1) serving at governmental and private hospitals of the Qassim region during the COVID-19 pandemic, and (2) consented to participate in the study. The healthcare providers and administrator staff were not approached for this study based on the exclusion criteria of not providing consent to participate in the study. Recruitment of participants was continued until we reached data saturation.

#### Consent process

**Figure 1:**
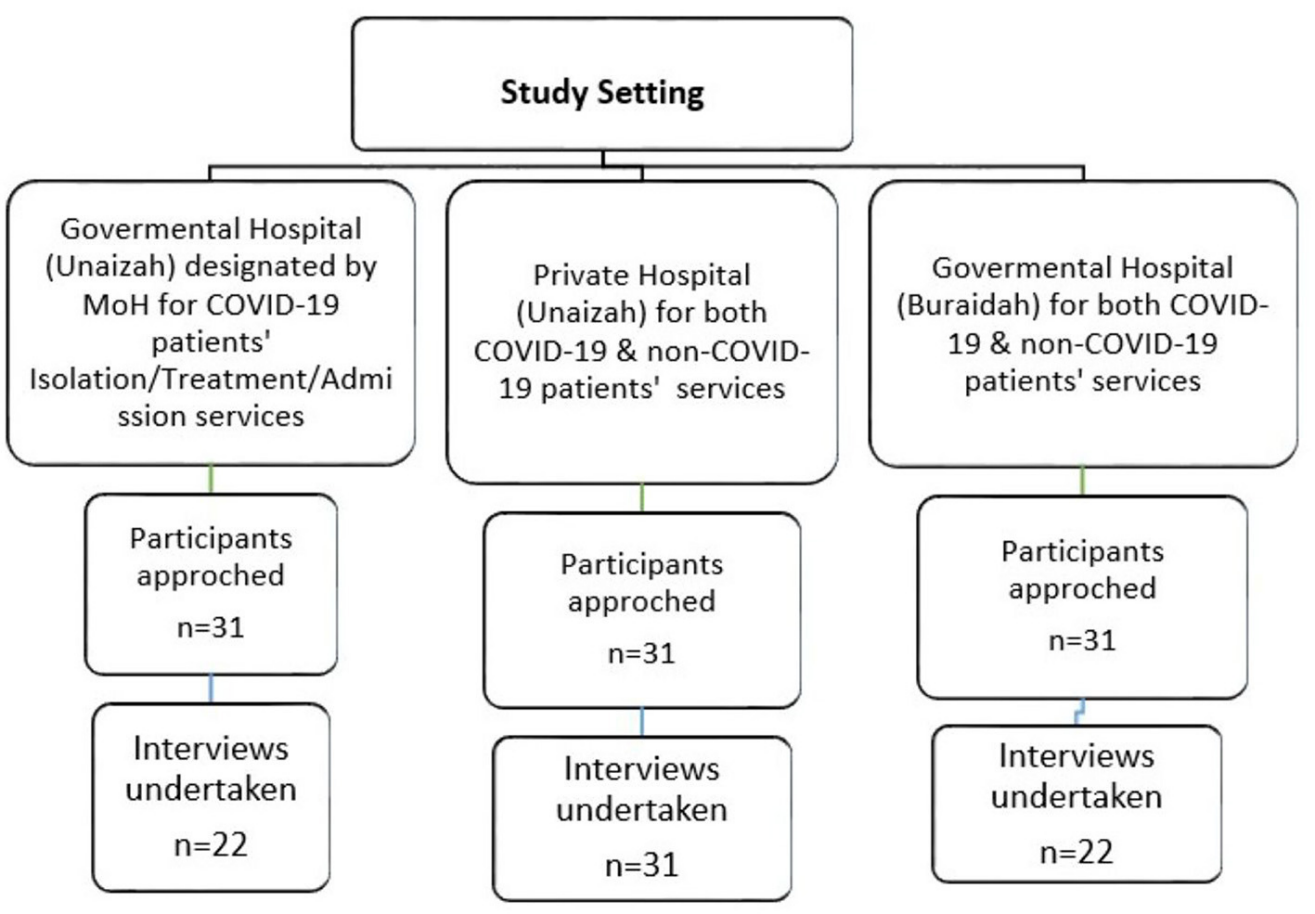
Flow diagram for recruitment.

A total of 93 potential participants (from all three hospitals) were initially approached face to face by three members of the research team, who introduced them to the background of the study. If the participants expressed interest and consented to join the study, they were asked to meet with the other researchers, and a mutually agreed date/time and place were arranged to conduct the interview. They were informed that the anonymity of the statements in the transcripts and final report would be ensured along with the security of the data collected (see Figure 1).

#### Discussion guide and interviews

The in-depth interviews were based on a semi-structured, open-ended interview guide developed by the two authors of this study in consultation with subject experts. To optimize the final interview guide and technique, this was piloted with three eligible participants who were not included in the final analysis. The resulting guide was revised and approved by all the authors. It consisted of open-ended questions that allowed the participants to discuss as many elements as possible. Interview guide included four sets of questions (1) participants' information including age, gender, occupation, nationality, years of experience, department/unit, and name of hospital (2) changes/new protocol implemented at their hospital during COVD-19 pandemic, taking into account general experience with changes and challenges and experience related to doctors' shifts/ rotation policy, referral policy, emergency department's protocol, medical/surgical appointments, criteria of admitting a patient, patients' attendant policy, level of satisfaction with the isolation ward specified for covid-19 patients (3) impact of adjustments/changes on them, including their family and daily life, and any history of psychiatrists' assistance (4) their experience and concerns for coming months.

Six members of the research team conducted all interviews. Two interviewers were there in each interview (one as moderator and one as observer). All interviewers had training in qualitative study processes to ensure consistency and credibility. Most of the interviews were conducted face-to-face in the office of each participant, but for those who had a very busy schedule, they were conducted through an online meeting app. All the participants followed the same interview guide, and moral reflexivity for researcher–participant relationship was maintained throughout all the interviews (32). The interviews were conducted from September to December 2021 (see Table 1). Each interview lasted between 45 and 70 min. During the interviews with the participants, the researchers took notes in their local language (Arabic) to retain a literal sense of the responses. The notes were translated and expanded after each interview. However, the non-Saudi participants (non-Arabic speakers) preferred interviews in English (see Table 2).

**Table 1:** Hospital characteristics (*n* = 3).

**Hospital**	**KSH (*****n** **=*** **22)**		**KFSH (*****n** **=*** **22)**		**HNH (*****n** **=*** **31)**		**Total (*****n** **=*** **75)**	
**Specialty**	***n***	**%**	***n***	**%**	***n***	**%**	***n***	**%**
General surgery Physician Nurse	3 2 1	13.62	5 3 2	22.73	6 4 2	19.35	14	18.66
Gynae/Obs	1	4.55	-		1	3.23	2	2.67
Internal medicine Physician Nurse	4 2 2	18.18	4 3 1	18.18	3 2 1	9.68	11	14.67
Pediatrics Physician Nurse	1 1 -	4.55	-		3 2 1	9.68	4	5.33
Neurology	1	4.55	1	4.55	1	3.23	3	4.00
Psychiatry	1	4.55	1	4.55	1	3.23	3	4.00
Radiology	1	4.55	2	9.09	2	6.44	5	6.67
ER Physician Nurse	4 2 2	18.18	3 2 1	13.63	4 2 2	12.90	11	14.67
ICU Physician Nurse	2 1 1	9.09	2 1 1	9.09	4 2 2	12.90	8	10.67
Laboratory Physician Technician	2 - 2	9.09	2 1 1	9.09	3 - 3	9.68	7	9.33
Manager/administrator	2	9.09	2	9.09	3	9.68	7	9.33

**Table 2:** Respondent characteristics (*n* = 75).

		**KSH (*n =* 22)**	**KFSH (*n =* 22)**	**HNH (*n =* 31)**	**Total**
Age (years)	< 30	1	3	5	9
	31–40	10	13	12	35
	41–50	9	4	10	23
	51–60	1	1	4	6
	>60	1	1	0	2
Gender	Male	18	15	16	49
	Female	4	7	15	26
Nationality	Saudi	12	12	7	31
	Non-Saudi	10	10	24	44
Occupation	Doctor/physician	9	10	12	31
	Surgeon	3	3	5	11
	Nurse	6	5	8	19
	Lab technician	2	2	3	7
	Manager/administrator	2	2	3	7
Experience (years)	1–5	2	2	7	11
	6–15	15	14	13	42
	16–25	3	4	10	17
	>25	2	2	1	5

#### Thematic analysis

Each interview was transcribed verbatim in either the local language (Arabic) or English. All transcripts were analyzed using the thematic analysis technique developed by Braun (33), Kiger (34), and Mayring (35). Initial notes were documented, and interpretations of each transcript were conducted by reading and reviewing the data. We adopted the inductive process for the exploration and assignment of codes to each meaningful sentence, after which similar codes were gathered in overarching subthemes through the joint effort of all authors. To gain first-hand experience of the participants' words and to obtain richer interpretations, nine transcripts (20%) were independently analyzed by the three authors. The initial coding was subsequently reviewed and compared by the remaining authors. The final grouping of similar subthemes under a main theme was then conducted. The collected data were reviewed and refined until all the authors agreed to a representative coding scheme, along with the main themes and subthemes.

#### Ethical considerations

The study was conducted following the Declaration of Helsinki and approved by the Regional Research Ethics Committee in Qassim province (Reference No. 607444162). Informed consent of the participants was obtained.

## Results

A total of 75 interviews were conducted using the developed semi-structured in-depth interview guideline (Table 1). Out of 75, more than half (65%, *n* = 49) of the participants were male. Almost half (47%, *n* = 35) belonged to the 31–40 age group. The majority of the participants were doctors (41%, *n* = 31), non-Saudi (59%, *n* = 44), and had work experience of 6–15 years (56%, *n* = 42), as shown in Table 2. The participants' responses can be grouped into four main themes. (1) changes in hospital policies and procedures, (2) workforce management, (3) the well-being of the hospital workforce, and (4) apprehensions and expectations of the hospital workforce. Selected quotes for each theme are presented in this section.

### Theme 1: changes in hospital policies and procedures

Policy changes and newly implemented procedures, as explained by the respondents of our study, are discussed under the four subthemes discussed here, including (a) administrative decisions, (b) visual triage (VT) and isolation rooms, (c) urgent referral networks, (d) hospital admissions, and (e) patient flow in different hospital units.

#### Administrative decisions

Our participants helped us discern the implemented policies during the current pandemic and explained that all hospitals in the country followed the Saudi MoH Protocol for Patients Suspected of/Confirmed with COVID-19. They reported that the Saudi MoH constantly updated its guidelines with evidence-based information. The protocol includes Ministry-approved manuals and instructions for healthcare workers dealing with COVID-19 patients in their vicinities. A hospital administrator shared the following information:

“Well, we had a very hard time when the pandemic was declared by the WHO, and the Ministry of Health's COVID 19 Protocol was changing according to national and international standards. I remember that we were getting the MoH's circulars very frequently. All OPDs were stopped, and all healthcare workers were deployed on an as-needed basis in different units of the hospital.” (A1)

Another administrator who worked during this time of the crisis stated:

“It is dealt with by following the standardized national prioritization policy of the Ministry of Health, which includes visual triage, and the cases are divided on the basis of their presentation.” (A2)

A new protocol included a number of changes to ensure timely intervention. As one governmental hospital physician described:

“I'd say new protocol covers all essential measures. There is even a checklist for doctors before they enter the hospital. The newly implemented protocol includes triage service/screening for all, isolation room, use of HEPA filters and personal protection equipment (PPE), the no visitor policy, safe distance, no OPD, and no elective surgery. Initially, people were very afraid, and there were fewer patients who visited the emergency department (ER), but now they're well oriented with the precautions. There are changes in the dates and times of appointments according to the new system and policy, but the care remains the same. This is because the working hours for the healthcare staff increased from 8 hours to 12 hours per day.” (P1)

An ER nurse added her perspective as follows:

“Actually, a lot of changes in infrastructure were made. The hospital administration upgraded the infection control unit and improved its sanitization measures. Over and above that, ER services were remodeled with the establishment of visual triage. Additionally, the number of beds was increased for infectious patients and high-risk patients in the isolation department.” (N1)

Infection control measures play a pivotal role in infection crisis situations. Related to this, an infection control manager expressed his contentment regarding the deployment of committed personnel for this important task:

“I'm very satisfied with our infection control measures. We have a monthly check-up at our hospital, and third-party infection control teams also visit frequently to ensure the quality of our implemented measures. We arrange regular workshops for the adequate training of our staff to use PPE properly and implement infection control recommendations, including wearing facemasks, hand washing, gown disposal, social distancing, and other evidence-based rules.” (A3)

The head of the nursing staff had similar sentiments:

“Well, it was initially a very difficult period because the hospital administration conducted a lot of meetings, and we were also attending lectures, live demonstrations, and awareness sessions through Zoom, as arranged by the MOH through infection control and the quality control units of our hospital. We were literally overwhelmed with that stuff. There was a lot to process that time, but now I feel lucky to have all those resources and the updated awareness, as it enabled us to combat the COVID-19 situation.” (N2)

#### VT and isolation rooms

The MoH implemented a compulsory policy involving the use of a VT scoring system in all hospitals for the early identification of COVID-19 patients. This system was explained by an ER department physician:

“The emergency department has established a special visual triage area for all patients who want to enter the hospital. If a patient has fever or any respiratory problem and has a score equal to or more than 4 in the visual triage for acute respiratory infection, then he/she is admitted in the isolation room.” (P2)

Isolation rooms in all governmental and private hospitals were also upgraded. A duty physician in the isolation department provided the following details:

“All isolation rooms are equipped with high efficiency particulate air (HEPA) filters. A separate medical team, including an emergency doctor with specified staff, deals with isolation cases, and each patient has a private nurse there. The nursing staff working in the isolation rooms are provided with a separate accommodation. They have separate pathways to enter isolation rooms and a separate duty roster to follow. After being admitted to the isolation department, a patient's COVID-19 status is confirmed by a pharyngeal swab.” (P3)

#### Urgent referral networks

A physician working at a designated COVID-19 center stated that if a patient suspected of having COVID-19 was admitted to a private hospital or any hospital not designated for the isolation and treatment of COVID-19 patients by the MoH, then he/she must be referred to a designated hospital upon confirmation:

“Our hospital is a designated COVID-19 center. So, we are receiving only COVID-19 cases from all hospitals in the Qassim Region. Likewise, whenever we come across any cases that are neither suspected (visual triage score of less than 4) nor confirmed COVID-19 cases but require admission for stabilization, then we refer them to a non-COVID-19 center/hospital allocated by the MoH, where they are admitted into the ICU/ward/isolation as per the requirements.” (P4)

#### Hospital admission

##### New criteria

An administrator specializing in therapeutic services at the MoH-designated hospital, who heads the provision of intensive therapy to patients, sees the new admission protocol as an important part of the fight against COVID-19. This is what he has to say regarding the implemented changes:

“We implemented a separate protocol for COVID-19 patients. To receive symptomatic patients by wards through the ER triage, a radical change in the admission protocols was activated, followed by a pharyngeal swab, and a verification of whether a patient was infected with COVID-19. The patients and staff were kept apart within the departments. Apart from severe COVID-19 patients, the MoH's criteria for admission during this pandemic include emergency cases due to road traffic accidents and childbirth cases.” (A4)

##### Policy for visitors/escorts

Visitor/companion policy for patient care partners during the pandemic was revised to protect the staff, patients, and all those who accessed hospitals to seek services. A ward nurse explained the revised policy as follows:

“According to the new protocol, visitors are almost forbidden until now. A patient's companion is also prohibited, but there are a few exceptions, such as those accompanying elderly and disabled patients, who are evaluated on a case-by-case basis.” (N3)

An ICU nurse further stated:

“As no companions or visitors are allowed, for critical cases, we communicate with the families daily and inform them about their patient's progress through the social service team in the hospital.” (N4)

Other details about the policy were communicated by a pediatrician:

“We consider a mother and child as one unit in case a child is admitted to our hospital. The mothers are instructed to remain inside the patients' room at all times. Standard precautions are taken by both, and they are designated for home quarantine upon discharge.” (P5)

#### Patient flow in different hospital units

Another emergent subtheme involved patient flow in different units of the hospitals. This subsection describes only those units that either had a distinguished role or had some new criteria or policy during the pandemic.

##### Outpatient department

From the interviews, it was quite evident that, at the start of the pandemic, many healthcare services, including non-urgent OPD appointments, were either suspended, postponed, or reduced in scale and scope to deal with COVID-19 infections. Participants playing various roles responded in similar ways:

“All the OPD clinics were suspended, and we were receiving only emergency cases since the pandemic was declared in the kingdom. Other changes include a reduction in the number of appointments and postponement of non-emergency appointments. All dental, dermal, and elective clinics were also stopped (except in medical emergencies).”

##### Radiology unit

Radiology units became particularly important after the clinical picture of COVID-19 became clearer in mid-2020, and the diagnosis of COVID-19 was linked with radiological findings of respiratory systems. Hence, there were some essential adjustments in this unit of hospitals to increase the efficiency of service delivery, as described by a radiologist:

“In our unit, radiologists and technicians are divided into two teams. These teams are alternately rotated between COVID-19 and non-COVID-19 sections for two consecutive weeks. The suspected or confirmed COVID-19 patients have to undergo an x-ray procedure via a portable machine, while the non-suspected and COVID-19 negative patients can go directly to the radiology department (non-COVID-19 sections).” (P6)

##### Operating rooms

Hospital managers and other participants serving in surgical units elucidated the distinctive challenges faced by the surgical workforce at hospitals compared to those with nonsurgical specialties. One of the considerable challenges faced by surgical units was how to safely stop non-urgent surgeries. Alongside the ramp-down in the operating rooms, a new strategy also needed to restructure how personnel should be deployed to provide care for COVID-19 patients. This unique task was accomplished by categorizing the surgical procedures, which was described in the following words by a hospital assistant manager serving at the MoH's designated COVID-19 hospital:

“All elective surgical procedures are postponed to maximize care for patients with COVID-19, and, at the same time, outpatient clinics are completely stopped to minimize risks to non-infected patients. We divided the essential surgeries into three categories. The first category includes urgent cases, the second includes cases of accidents and child delivery, and the third includes any important but non-urgent surgery. We refer the third category to other hospitals, as we can only attend to critical cases that may result in complications.” (A5)

##### Intensive care units

A practical example of teamwork can be seen in hospitals' ICUs, where doctors and paramedical staff from many other disciplines, including surgeons, internists, anesthesiologists, and others, were added to the teams to achieve the best possible results. As an administrator of patients' affairs at the region's busiest hospital designated for COVID-19 explained:

“We are receiving COVID-19 cases referred from different hospitals because King-Saud Hospital is one of the regional COVID-19 centers. We've expanded the ICU for critical cases, including gynecological and pediatric critical cases. There was a point in time when the number of referred COVID-19 patients was gradually increasing, and so were our ICU teams, as teamwork proved to be essential in dealing with this pandemic.” (A6)

##### Obstetrics and gynecology units

The public authority (MoH) instructed the cancellation of all non-essential medical and surgical activities in all hospitals. Hence, units/departments revisited their activities and suspended non-urgent procedures. However, the gynecology and obstetrics units faced the unique challenge of maintaining the provision of safe care for pregnant women and reducing/suspending other non-emergency activities. A consultant serving the OB/GYNE unit explained this professional obligation by comparing it with other healthcare roles:

“Well, ours is the only specialty that has to continue most of its activities, and our responsibility became twofold during this situation. Even though we suspended the elective gynecological cases and shifted some of the activities to virtual consultations, the follow-up appointments of pregnant women required physical examination, ultrasonography, and monitoring blood pressure and weight, among others. So, obstetric cases can't be attended through video consultations. Furthermore, we've dedicated a separate ultrasound machine and separate CTG machine for the COVID-19 patients.” (P7)

Another gynecologist elucidated in her interview that a special COVID unit was allocated for pregnant and postpartum women, which implied a separate roster that included consultants, residents, childcare assistants, and nurses. She further explained that they have a special policy in place for the surveillance of neonates delivered by COVID-19-positive mothers:

“We separate the babies born by COVID-19-positive mothers and keep them in a segregated area, which is especially designated for monitoring these babies.” (P8)

The gynecologist also brought to light an important issue regarding COVID-19 vaccination. When it was initiated in the early part of 2021 in KSA, tension remained high in this specific OB/GYNE unit, because the pregnant and post-partum women were hesitant to receive the vaccine:

“Lactating mothers and pregnant ladies were hesitant to receive the COVID-19 vaccine, and had doubts in their mind. These women were even reluctant to increase their awareness regarding their vaccination.” (P9)

### Theme 2: workforce management

The participants explained that a clear and planned approach was adopted to manage the hospital staff in the sample hospitals. This approach had a dual purpose: ensuring physical and mental protection and creating an environment that enabled them to perform their jobs well. Hospital workforce management is discussed under two subthemes: (a) rapid response teams and (b) digital platforms and video consultations.

#### Rapid response teams

Several doctors commented that to deal with crisis situations, one must be able to work as part of a team. As a senior operational manager described:

“Well, I am going to be very precise here, and try my best to make you understand a quick summary of what we did in our hospital. We've distributed the whole staff (including doctors, nurses, housekeepers, etc.) into two teams. Each team has to work for two weeks and then has two weeks off. If a member of one team gets infected, then this deficiency is compensated from the other team.” (A7)

The duty rosters and hours were updated in all hospitals in the region for a more effective and timely response. The staff seemed to exhibit an eagerness to engage with the patients. According to a physician, this eagerness contributed to their willingness to work efficiently:

“At that time, the most important change was our shift schedule and our eagerness to serve. The duty timing was increased from 8 to 12 hours, and some of us even worked more than 12 hours or up to 17 hours per day. Half of the staff was working, and the other half was resting, so that if anyone of them is infected, then he/she does not transmit the infection to other team members.” (P10)

#### Digital platforms and video consultations

The Saudi MoH has launched some informatics applications for the health sector during the COVID-19 pandemic in the country. The participants explained that these smartphone apps ensure the provision of preventative care and clinical instructions for home quarantine with daily follow-ups, tracking signs/symptoms of mild cases and their contacts, booking online appointments for COVID-19 clinics and diagnostic centers, and video consultations for non-urgent/non-COVID-19 cases. Some healthcare personnel thought that this was an opportunity to learn the application of such technologies in the healthcare sector:

“It was a very difficult period initially, but then I started realizing that not everything was negative, because there were many positive aspects as well, such as virtual clinics (online appointments and video consultations), which enhanced my online communication skills.” (P11)

Others believed that these services were helpful in reducing their workload and unnecessary exposure to viral infection. One internal medicine consultant expressed this view:

“I see digital services as altruistic. In my opinion, online consultation was one of the most useful services introduced during the COVID-19 pandemic last year. It was implemented in all clinics/specialties, as there was no OPD. Online home consultation includes health consultations and medicine delivery to the patients' homes.” (P12)

Another consultant serving at the OB/GYNE unit showed appreciation for technology:

“The best action by our hospital administration was the provision of an app (Medica Plus) on our laptops, which allowed us to monitor our admitted patients through our laptops.” (P13)

### Theme 3: well-being of the hospital workforce

The ongoing pandemic is an actual example of the healthcare workers' dedication to save lives. They are constantly exposed to several job-related challenges during this medical crisis, including physical and mental health issues. These health challenges are discussed separately under two subthemes: (a) physical and (b) psychological well-being.

#### Physical well-being

An alarming number of medical professionals contracted COVID-19, while saving the lives of their patients during this pandemic. Therefore, it became an issue of concern for the Saudi MoH. To ensure their safety, healthcare workers were provided with PPEs and were given priority in the KSA's vaccination program. Yet, they still faced a greater risk of infection compared to that of the general population. One internal medicine consultant, who was rotated for the duty schedule that covered a period of 14 days, shared her experience as follows:

“After completing my duty period, I had to go through 14 days of isolation and a COVID-19 test, and that was how I came to know that I tested positive for the virus. I was very surprised because I contracted COVID-19 even after receiving my 1st vaccine dose.” (P14)

An ICU nurse narrated how she felt after contracting the infection from the hospital:

“I tested positive for COVID-19 but had no symptoms. I was fine physically and mentally during my quarantine period, but I was concerned for every person who was in contact with me during the last few weeks.” (N5)

#### Psychological well-being

##### Psychological impact

There is a wide range of mental health risks faced by healthcare professionals due to their unprecedented task of juggling their own safety and that of their families, while minimizing the suffering of their patients. A few of the major concerns communicated by the interviewees were exhaustion due to long working hours and family safety issues. One of the native internal medicine nurses explained it as follows:

“I was very stressed because it's a new disease and we have to deal with it carefully. We worked for 12–14 hours a day during the pandemic, and my life was totally changed after a few of my coworkers contracted COVID-19.” (N6)

A similar concern was expressed by a doctor working in an emergency medicine unit:

“I didn't have work-related stress or depression, but the only thing that haunted me was fear of transmitting the virus to my family.” (P15)

A foreign gynecologist also revealed her stress-related experience:

“Honestly speaking, I was scared after realizing that I was stuck in the middle of a situation where my colleagues were contracting COVID-19 infection and some of my family members in Egypt were suffering from the virus too. Then, the stress became uncontrollable for me when I learned that my mother contracted COVID-19 after receiving her first dose of COVID-19 vaccine. To make the situation even worse, I discovered that I was pregnant. Finally, I delivered my baby during the COVID-19 pandemic, despite all these circumstances. It was very stressful. I cannot forget it.” (P16)

Other healthcare workers also shared similar experiences:

“I had a severe episode of depression after I contracted COVID-19 and was admitted to the ICU. I felt helpless and very anxious for my family at that time. My anxiety was relieved when my family received their vaccinations.”

“I had replacement duties to compensate for the absence of my infected colleagues. Changes in shifts and increased workloads affected my psychological well-being in a negative way. I faced severe insomnia and depression.”

“Our social lives have changed. Even though I changed my clothes and shoes before leaving the hospital every day, I was afraid of transmitting the virus to the people around me.”

“I personally felt like we were not living a normal life. Sometimes, I didn't have time to eat. The last few months were so difficult to cope with that I needed psychiatric assistance. That was because my husband and I became infected with COVID-19 at the same time and had nobody to take care of our children.”

##### Self-help interventions

All doctors and paramedics in our sample reported emotional disturbances of varying degrees and the need to manage their stress. The majority of these respondents described the work itself with delight and vehemently disagreed when they were asked about the widespread opinion that healthcare workers needed psychiatric assistance to deal with their workloads. A common response was:

“In my case, I didn't need formal or informal counseling or psychiatric assistance at any stage of the pandemic.”

Some of them even responded as follows:

“I am proud to be a healer, and I feel an internal satisfaction because I am an organized person who follows the standard protocols by the Ministry and the guidelines issued by the Infection Control Department.”

“I didn't have stress; everything was under control. I used to go to hotels designated as quarantine facilities and talked a lot with my colleague nurses every day after I finished my work at the hospital.”

##### Psychological/emotional support

Most of the respondents cited their colleagues and friends as determinants of their sustained mental health:

“No, I never consulted a psychiatrist, but sometimes I talked to my friends working in the medical field and found it refreshing.”

“I think accepting the situation and counseling a work partner are sometimes very useful strategies. I tried both of them and found them effective for me.”

Others shared that changing their routines to ensure that they had resources outside of their work enabled them to remain enthusiastic about their jobs. As one surgeon stated:

“I started playing racket with my colleagues, reading books, and talking to my family more than we used to do before the pandemic, which helped me enormously in controlling my stress.” (P17)

##### Psychiatric assistance

A few doctors and nurses had taken time out from their work when they realized that they were close to burning out. They also consulted psychiatrists for their insomnia, anxiety, and depressive symptoms, saying that:

“It affected everyone around me. I experienced panic attacks, and my sleep cycle was disturbed. I was depressed. On a scale of severity from 1–10, I'd rate myself as 9! I was afraid of the COVID-19 vaccine, too. At that time, I could not even go outside for a change. I felt helpless and stuck. Then, I talked to my husband, and he decided to bring me to a psychiatrist.”

“I was anxious for my three-month-old son, felt afraid most of the time, and then gradually noticed that I was depressed. Then I attended a few counseling sessions by my psychiatrist colleague and felt relieved.”

“I consulted a psychiatrist after I became infected with COVID-19 because I was unable to sleep (insomnia) for three days continuously. He diagnosed me as having post-traumatic stress syndrome and prescribed alprazolam for me.”

“I needed informal counseling from a psychiatrist friend to reduce the fear of contracting COVID-19, and I found that psychological counseling is pretty good in reducing stress and finding the right way to deal with the current situation.”

From the perspective of psychiatrists, there is an overall increased rate of consultations during the pandemic, not only by their colleagues working in the same hospitals, but also by the general population:

“Cases of obsession increased during the current situation. I have to conduct many counseling sessions with my colleagues and outpatients. The topmost condition is obsessive-compulsive disorder (OCD), but other conditions include insomnia, panic attacks, anxiety, and depression.” (P18)

A psychiatrist pointed out another interesting aspect:

“I can tell you that people with pre-existing psychotic conditions, such as schizophrenia and bipolar disorder, were affected more in terms of severity, as compared to neurotic conditions, such as depression. Another noticeable factor was the rate of cases of OCD, which almost tripled, both among the general population and healthcare workers during the current pandemic.” (P19)

### Theme 4: expectations and apprehensions of the hospital workforce

Our study participants across a wide range of positions and jobs spoke about their concerns in the upcoming months as follows:

“I am afraid that COVID-19 will spread more in the coming months because we are approaching the winter season, and the precautions have been greatly reduced among the general public, especially the lack of social distancing and failure to wear facemasks.”

An ER doctor suspected that this pandemic might even last forever:

“Harsh, but the truth is that COVID-19 will never end. We have to live with it, or maybe with some of its variants.” (P20)

Some of the participants were worried about the new variant—a worry expressed by a lab assistant who said that:

“I think cases are decreasing all around the world, and I know it sounds fatuous, but I feel like the pandemic will reoccur with a new wave due to the Omicron variant. It's difficult to know whether the vaccine will help against new variants or not, but most probably these vaccines will not protect us from these new variants of SARS-CoV-2.” (L1)

Some also doubted the efficacy of the vaccines in varying degrees:

“I'm afraid, those days will come back with all the stress. I don't think the vaccine will be fully protective against the upcoming variants. Its effectiveness will decrease with time if it is not boosted with another dose, and I am afraid of the season in which the virus increases.”

“I think all of us know that the vaccine may not be fully protective, but we have been dealing with this situation for almost two years, and we already know how to live with it in the coming years. I am not scared. I'm ok with online classes for my children and expect that it may become seasonal infection like influenza.”

At the same time, others were concerned about the increase in cases of mental disorders:

“God willing, it will not continue and will end within a couple of months, but if the pandemic continues, then there will be a sharp rise in mental illnesses, such as depression and anxiety.”

A pediatric consultant voiced concerns about children's health in the coming season:

“There was a moment in time last year when I thought we'll never be able to understand this virus completely, but now I feel good that we have sufficient information to deal with it. As it is a new disease, we are still learning about the organism itself, and the most important finding about COVID-19 is that we've started noticing a significant reduction in other viral infection cases, such as influenza and bronchiolitis, among children due to strict precautions. But now, as we are resuming back to normal, the common infectious cases are also returning gradually to their usual numbers among children.” (P21)

Cases of COVID-19 caused by the Omicron variant were first detected in South Africa and were eventually reported in other parts of the world. Transmissibility aside, the key source of positivity for some HCWs appeared to be the use of vaccines, and it seemed to make them feel that they were protected:

“The new Omicron variant started spreading and may lead to the continuation of this pandemic, but I think it will be controlled well now, because the MoH already knows how to cope with the pandemic and has already made preparations. The majority of the kingdom's population is vaccinated. Also, I think people are well aware of how to protect themselves and their loved ones. So, I think, it won't be that aggressive as before, and I see a glimmer of hope in the form of vaccines.”

“I think this Omicron is not as virulent as the Delta variant. Yeah! It is spreading fast, but most of us are already vaccinated and protected from this new variant. Still, everything is in the hands of God. We cannot predict the future, but we can have the best expectations.”

“Our hospital administration is alerting us to maintain the same precautions like using masks and social distancing. Of course, COVID-19 is a droplet infection, and this variant is spread by similar routes. So, we have to be careful and ready to deal with it. I'm not sure, but I think the new variant (Omicron) is weak, and the vaccines can minimize its spread. I believe this pandemic will end soon.”

Conversely, there were some healthcare workers who expressed their view in a hopeful and optimistic way by revealing their expectations as follows:

“In the beginning, there was a lack of information and PPEs, but after a month, PPEs were provided to us. Now, I'm satisfied as things are getting better. Mass vaccination is already being done worldwide, along with the continuation of the strict implementation of precautions, including washing hands and wearing face masks.”

A nurse seemed satisfied with the MoH's timely measures, saying that:

“If I compare the KSA with the other countries, the KSA implemented a lot of strict measures and timely policies to contain the virus, and I really appreciate this. So, I'm hopeful that Omicron will not spread here in the kingdom like in other parts of the world.” (N7)

Some felt that the global situation was normalizing gradually:

“The COVID-19 virus is now getting weak, and for the first time in over two years, I feel like the pandemic has already ended, because a lot of things are getting better day by day and resuming normality.”

## Discussion

To the best of our knowledge, this is the first study to conduct in-depth interviews with a sample of respondents in all roles and professions to explore the nature of hospital adjustments and the concerns of healthcare providers and administrators. These respondents have served for sustained periods in different hospitals throughout the Qassim Region, KSA, during the COVID-19 pandemic. The study results have relevance for both national and international healthcare systems.

First, this study provides an overview of new polices and modifications in the pre-existing healthcare system of KSA, along with the perspectives of healthcare providers serving in those settings during the pandemic. A number of approaches were employed by policymakers around the world to scale up their services in healthcare systems in response to the COVID-19 pandemic. For instance, within 2 weeks, China constructed two new special field hospitals for the treatment of COVID-19 patients in the early months of 2020 (36). Meanwhile, in some countries, the primary healthcare systems became receptive points in the form of COVID-19 triage, and acute services were also made available at these centers (37). However, the KSA employed a “whole-of-government” approach to strengthen their healthcare facilities by referring to their previous experiences of a similar viral infection (i.e., MERS) (20). The evidence-based strategies were translated into policies, and more than 25 regional hospitals were designated for COVID-19 cases through the effective integration of healthcare delivery using public health and technology tools (15). Our study explored a detailed account of processes followed by hospitals and verified that an organized national treatment protocol was put in place for suspected and confirmed cases of COVID-19 by the Saudi MoH (20). The protocols were regularly updated to meet international standards and included medical care for all COVID-19 cases, ranging from mild to severe (38). The MoH deployed trained monitoring teams to evaluate compliance with new protocols to ensure their effective implementation throughout the kingdom (15). The MoH-designated regional hospitals assigned patients to the isolation ward on the basis of a VT system, which included a checklist for the initial screening of patients at the entrance of each hospital (39). All of those patients were then swabbed upon admission to the isolation ward and were subsequently transferred to either the COVID-19 or non-COVID-19 units based on their lab test results (40). Separate transits and hallways were established in the physical spaces of the hospital to access the COVID-19 units, not only for the safety of COVID-19-positive patients but also for the staff who were taking care of them (41). There was a strict code for the medical staff of COVID-19 units, which included the use of PPEs, gloves, masks, gowns, and face shields (42).

VT had two sections, with a total of nine items. Section 1 is related to the risk of exposure to SARS-CoV-2, including a history of travel abroad in the past 14 days, contact with a confirmed case of COVID-19 in the last 14 days prior to onset of symptoms, or whether someone is working in a healthcare facility. Section 2 is about a patient presenting signs/symptoms, including fever or a recent history of fever, cough (new or worsening), shortness of breath (new or worsening), headache, sore throat, rhinorrhea, nausea, vomiting, and/or diarrhea; immunocompromised status; and a history of chronic renal failure and CAD/heart failure. If a patient's score is ≥4, then he/she is isolated and made to undergo a reverse-transcriptase-PCR (RT-PCR) test to rule out COVID-19 (39). The aforementioned regional governmental hospitals were dedicated to the treatment of COVID-19 patients, while other hospitals, including private hospitals, were accommodating most of the non-COVID-19 patients (15). A network for referrals was established by the MoH among all hospitals at the regional level, and almost all COVID-19 cases were referred to designated hospitals. At the same time, non-COVID-19 cases were referred to other hospitals by the COVID-19 designated hospitals for the strategic management of these cases (43). Governmental hospitals are best utilized during the ongoing pandemic by countries, such as China in their initial phase, and the UK where the National Health Service implemented interventions similar to the KSA government (44, 45). There was a partial preservation of medical activities, such as cases of child delivery, accidents, and some urgent ones, while all elective non-urgent activities, including surgeries, were completely stopped in all hospitals (15). The Saudi government's policies not only ensured easier access to hospitals and an organized management of COVID-19 patients, but also effectively reallocated the hospital staff in different units while also controlling urgent referrals. These findings are analogous to a study by Magro et al., who reviewed the primary challenges faced by a tertiary healthcare hospital in Italy during the early stage of the pandemic (46), and to a recent study in Italy by Carenzo et al. (8).

There was a significant role of digital health services in KSA during pandemic. Around 89% of the population (30,260,000 people) in the KSA are Internet users, and a vast majority (96% of the population) use smartphones, tablets, desktop computers, and laptop computers (47). When the COVID-19 pandemic broke out, the MoH activated two already existing mobile and web-based apps, Mawid (“appointment”) and Sehhaty (“my health”), to help patients book appointments at COVID-19 clinics and drive-through testing sites (48–50). Another smartphone application called Tetamman (“rest assured”) was launched to provide guidelines for home isolation with daily follow-ups and tracing of contacts (51).

Other apps included Tawakkalna and Tabaud. Tawakkalna (“we trust”), which recorded seven million users by the end of August 2020, was used for monitoring and reporting of suspected/confirmed cases, thus breaking the chain of infection, while Tabaud (means “distancing”), which started with 15,000 users, alerted people who came in contact with confirmed COVID-19 cases (52, 53). Moreover, video consultations, tele-clinics, and tele-radiology were employed to control the spread of COVID-19 (15). Telemedicine served around five million users, while the Tetamman app served about one and a half million users from April to August 2020 (54). All of these electronic platforms effectively provide healthcare and epidemiological surveillance during this ongoing pandemic, similar to many other countries with existing digital systems for the provision of various healthcare functions (55). For instance, the US and Singapore use chatbots and telehealth to provide information and remote triage of care to patients within their homes (56, 57). Likewise, the UK launched a COVID-19 tracker app for the timely detection of symptoms for further interventions (58). India also implemented telemedicine services to replace face-to-face checkups (59). Furthermore, the use of artificial intelligence (AI) for COVID-19 was introduced by Taiwan during this pandemic (60).

As COVID-19 cases surged globally, a proportionate increase in SARS-CoV-2 transmission was observed among healthcare workers all around the world, mainly due to their regular exposure to patients and fellow workers while at the hospitals (61, 62). Five important decisions were made in KSA to optimize the delivery of care and minimize interactions between healthcare providers, between them and their patients, and between them and their own family members. The first decision was to reallocate the health workforce in the emergency units and ICUs, while the second decision was to equip them with upgraded knowledge of how to contain the crisis. The third decision was related to providing them with appropriate PPEs to ensure their protection from infection, while the fourth decision was to divide them into two teams working on rotation to reduce their physical fatigue and interaction. The final decision was to change their working hours per day to ensure the effective management of the cases. Our findings are strengthened by studies, which concluded that more efficient results can be achieved through teamwork in healthcare and can prevent healthcare providers' voluntary turnover intention (63, 64). Another study also supports our results, indicating that health personnel rotations and shift schedules are efficient strategies for optimum patient care (44).

Psychological disturbances, such as self-reported stress, insomnia, and some psychiatric disorders (e.g., depression, anxiety, PTSD, and panic disorder), were prevalent among the study participants during this major crisis situation. Our findings are in line with a meta-analysis of 38 studies published in 2020 about mental health disorders among healthcare providers. The results showed the highest pooled prevalence of PTSD (49%), which is commonly manifested as insomnia and stress, while anxiety (40%) showed the second highest proportion, followed by depression (37%) and distress (37%) (65).

However, for mental health problems in the general population, the pandemic has not only impacted pre-existing mental health problems but has also led to the new onset of psychiatric conditions among the general population (66). The pre-existing psychotic disorders, such as schizophrenia, bipolar disorders, and major depressive disorders, more than neurotic disorders, such as depression and anxiety, worsen among the general population. This finding is in agreement with the expected impact of the COVID-19 pandemic reported in some studies, which indicated that relapses among schizophrenic and bipolar disorder patients are likely during this crisis due to the stress associated with the COVID-19 outbreak (67, 68). There was a sharp increase in cases of OCD, which lies on the continuum between psychotic and neurotic disorders, among healthcare providers and the general population (69). The main reason for this might be the exacerbation of obsessions about contamination and washing compulsions due to advice on the improvement of personal hygienic measures from international authorities, including the WHO (70). A similar finding was reported by a recent study in Canada, which concluded that OCD cases are on the rise during the current pandemic (71). A systemic review also reported worsening OCD symptoms, while an Egyptian study found a prevalence of OCD (28.2%) among the general population and healthcare workers (72, 73).

In our study, the topmost contributing factor for psychiatric disorders among the respondents was the fear of spreading the virus to their family members. This finding is reinforced by a recent qualitative study in Qatar (74). Other factors were the lockdowns imposed by the government, which jeopardized normal daily routines and social rhythms, the uncertainty about the spread of the pandemic in the coming months, and recurring worries about contracting COVID-19 infection (75). Different coping mechanisms were adopted including self-help strategies by staying positive, sought emotional support from their family members, and psychiatric assistance. A recently published cross-sectional study also found analogous results, reporting that almost 70% of healthcare workers used “family support” and “positive thinking” as their coping methods during the current pandemic (76).

The endemicity of the COVID-19 infection is expected as an endpoint of this pandemic, and Omicron as a new SARS-CoV-2 variant of concern for a mixture of fatigue, *déjà vu*, and dread. The new SARS-CoV-2 variant, B.1.1.529, was reported to the WHO on November 24, 2021 by South African scientists. To date, evidence suggests that Omicron is more transmissible than the original strain and its Delta variant (77). Even though it is expected that current vaccines will provide protection against hospitalizations, severe illness, and deaths due to the Omicron variant, the importance of boosters should be emphasized, and more data should be gathered to ensure accurate conclusions about its severity, case fatality rate, chances of reinfection, and infection of fully vaccinated individuals (78).

This was a large-scale qualitative study with a large sample involving three large healthcare settings in the central region of KSA; hence, saturation was achieved with utmost clarity. Our research team sought to ensure the inclusion of a broad range of professional perspectives within the interviewed sample so that it reflected a diversity. Despite these strengths, our study has three limitations. We adopted a purposive sampling approach. Thus, it cannot be presumed that our findings are representative of all healthcare providers and administrative staff of all hospitals across the country. Secondly, busy schedule of some of our participants made us conduct some interviews online. Furthermore, transcripts were not returned to participants for comment or correction. However, we believe returning the transcripts may made them change their responses, which may attenuate the validity of the original opinion.

## Conclusion

This research revealed that participants were initially overwhelmed by new administrative policies and interventions but gradually accepted them optimistically. Numerous innovative interventions effectively reduced their physical workload and increased their productivity, but they remained significantly affected by a wide range of psychological disorders, with a high prevalence of obsessive-compulsive disorder. There were some concerns about the new SARS-CoV-2 variant, but the majority were optimistic. Participants used different coping strategies, such as self-help strategies by staying positive, sought emotional support from their family members, and psychiatric assistance to overcome the psychological challenges. We recommend these coping strategies to create positive working conditions for healthcare professional and administrative staff, and the planned use of technology and data, along with the cogeneration of healthcare services with contemporary entrants in the healthcare field to combat future pandemics successfully.

## Data availability statement

The original contributions presented in the study are included in the article/supplementary material, further inquiries can be directed to the corresponding author.

## Ethics statement

The study was performed following the Declaration of Helsinki. The study was approved by the Ethics Committee at Qassim Region (ID: 1443-409368). The patients/participants provided their written informed consent to participate in this study.

## Author contributions

OA-W, RK, and PS designed the study. MA, AFA, and YA acted as qualitative supervisors and organized the interviews. SA, AAA, IIA, HA, RA, and IAA conducted the interviews. Primary analysis was conducted by RK, OA-W, PS, and formally reviewed by MA, AFA, and YA. The first draft of this manuscript was produced by RK and OA-W, which was then reviewed and revised by all authors. All authors read and approved the final manuscript.
